# TCGA-TCIA Impact on Radiogenomics Cancer Research: A Systematic Review

**DOI:** 10.3390/ijms20236033

**Published:** 2019-11-29

**Authors:** Mario Zanfardino, Katia Pane, Peppino Mirabelli, Marco Salvatore, Monica Franzese

**Affiliations:** IRCCS SDN, Via E. Gianturco, 113, 80143 Naples, Italy; kpane@sdn-napoli.it (K.P.); pmirabelli@sdn-napoli.it (P.M.); direzionescientifica@sdn-napoli.it (M.S.); mfranzese@sdn-napoli.it (M.F.)

**Keywords:** radiogenomics, cancer diagnosis, TCIA, TCGA, radiomics, genomics

## Abstract

In the last decade, the development of radiogenomics research has produced a significant amount of papers describing relations between imaging features and several molecular ‘omic signatures arising from next-generation sequencing technology and their potential role in the integrated diagnostic field. The most vulnerable point of many of these studies lies in the poor number of involved patients. In this scenario, a leading role is played by The Cancer Genome Atlas (TCGA) and The Cancer Imaging Archive (TCIA), which make available, respectively, molecular ‘omic data and linked imaging data. In this review, we systematically collected and analyzed radiogenomic studies based on TCGA-TCIA data. We organized literature per tumor type and molecular ‘omic data in order to discuss salient imaging genomic associations and limitations of each study. Finally, we outlined the potential clinical impact of radiogenomics to improve the accuracy of diagnosis and the prediction of patient outcomes in oncology.

## 1. Introduction

The development of high-throughput technologies in biomedical research has led to the high availability of data and, as a reflection, to the development of new techniques and methods to analyze the amount of these data. In the field of cancer diagnosis and therapy, both molecular biology and imaging shall be a reference tool to better characterize tumor phenotype. In recent years, advances of both approaches and the promising results arising from the correlation of cancer imaging features (radiomics) with high-throughput data (or transcriptomic, proteomic, etc.) has led to the new research area known as “radiogenomics” [1]. The mainstay of radiogenomics is the potential to investigate the relationship between different types of data by using advanced computer science methods able to manage and analyze a very large number of variables arising from many acquisition modalities [2]. Different and complementary layers of information (i.e., radiomics, genomics, transcriptomics, proteomics) contribute to identifying more and often distinct aspects of a disease. Integrating all features, considering data from each level, can increase the capacity to predict patient clinical outcomes. We referred to each level as omic. In this context, some data integration and data processing challenges need to be addressed [3]. One of the most critical challenges regards the ‘omics data acquisition and the obstacle of missing data, which may occur due to data filtering or non-execution of a specific analysis on a subset of specimens originating from separate laboratories. For these reasons, it is often hard for a research group to collect a significant quantity of both radiomic and biological omic data. A solution to this kind of problem is the use of public databases for molecular and imaging data available for different biomedical research fields. Among them, are the National Institute of Mental Health (NIMH) Data Archive (NDA) [4], stores clinical/phenotypic, imaging, genomic, and other data from hundreds of thousands of research participants. Moreover, The Cancer Genome Atlas (TCGA), [5] in connection with The Cancer Imaging Archive (TCIA) [6], represents the largest data repository in cancer research. TCGA-TCIA data have become crucial support also for cancer radiogenomics studies, owing to their collections for several primary sites and a large amount of available data (over 20,000 primary cancer and matched normal samples crossing 33 cancer types). In this scenario, also the role of the database that contains non omic data should not be underestimated. An example is the Atlas of Genetics and Cytogenetics in Oncology and Hematology [7] that makes available relevant biomarkers for diagnosis or therapeutic targets as well as the clinical outcome of patients. These data can be combined into a radiogenomic framework, adding new knowledge in order to identify cancer genes pathways or characterize different phenotypes. This review provided an extensive overview of works (Table 1) based on TCGA-TCIA data and, consequently, defined the role of these databases in radiogenomics cancer research. To this purpose, we first grouped the studies based on the tumor type and involved molecular features. We also described some studies not properly definable as radiogenomic studies. Indeed, many “radiogenomics” studies are actually based on a few features and do not consider overall information from ‘omic experiments. Moreover, in several cases, the involved features cannot be considered as true ‘omics, as in the case of radiological parameters. Finally, we discussed the search strategy, articles selection, the current challenges of radiogenomics, and the common limitations of each study.

## 2. Results

According to our search strategy, many unequivocal radiogenomic studies arise from investigations performed on the brain (GBM—glioblastoma multiforme and LGG—low-grade glioma) and breast tumors (BRCA, breast cancer) (Table 1). For this reason, we organized these two large collections of studies in subsections based on tumor type along with the analyzed molecular features, respectively. In addition, we shed light on the clinical aspects investigated for both diseases. In particular, for GBM, we discussed cancer sub-groups classification (also based on gene expression data), relations between imaging features and gene expression data and mutation data and proteomic data. Instead, for breast cancer, we discussed associations between imaging features and cancer subtypes, correlations between imaging features and gene expression data, and relations between imaging features and multiple molecular omic features. Doing that, we found that each study pointed out to establish a relationship between imaging features and genomic signature to clarify several clinical-pathological characteristics, including diagnosis, tumor stage, treatment response, and clinical outcome. For example, early detection of breast cancer intrinsic molecular subtypes is crucial to predict the most effective therapeutic strategy; also, the breast patients’ prognosis can be different based on tumor aggressiveness. From a genomic point of view, our results indicated that transcriptomic profiles represented the leading omic data type used so far for the study of disease genotype. Indeed, aberrant gene expression between healthy individuals and patients is commonly used to identify a relationship between imaging features and genomic pathways. The general approaches used by each study and the principle identified relationships are summarized in Table 2.

### 2.1. Glioblastoma Multiforme (GBM) and Low-Grade Glioma (LGG)

**Figure 1:**
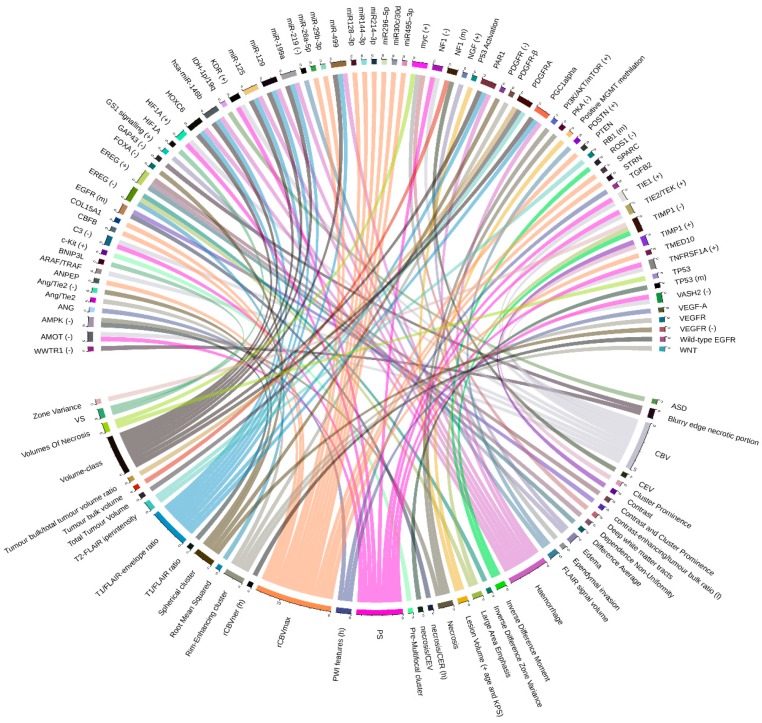
Radiogenomic associations in TCGA-TCIA GBM. Molecular omic features are represented on the top of the image, while imaging features are represented on the bottom. The arcs represent relations. (–) indicates a negative relation, (+) a positive relation, (m) mutation of the corresponding gene, (l) a low value of the corresponding feature, and (h) a high value. CER: Contrast-enhancing ratio, CEV: Contrast-enhancing volume, TCGA: The Cancer Genome Atlas, TCIA: The Cancer Imaging Archive, GBM: glioblastoma multiforme.

The overwhelming volume of literature within TCGA-TCIA radiogenomics studies has concerned brain tumors, such as the glioblastoma multiforme (GBM) and, at less extend, low-grade glioma (LGG). Glioblastoma is the most frequently occurring primary malignant brain tumor in the adult [8], and it is characterized by poor response to treatment. Some reasons are the histological and genetic intratumor heterogeneity and the consequent coexistence of different subpopulations of glioblastoma. Twenty-one radiogenomic studies were included for GBM and LGG (for a complete overview of radiogenomics in glioblastoma, see Kazerooni et al. [9]). The more detailed relations by the original articles are graphically represented in Figure 1.

#### 2.1.1. Cancer Sub-Groups Classification

The first extensive radiogenomic investigation using quantitative volumetrics features from magnetic resonance imaging (MRI) and gene/microRNA expression profiling in TCGA-GBM was performed by Zinn et al. [10]. They applied a novel diagnostic method to discriminate among molecular cancer subtypes and genomic correlates of cellular invasion patterns. A total of 78 treatment-naïve patients were equally split into three sub-groups (high, medium, and low) based on FLAIR (Fluid Attenuated Inversion Recovery) signal volume, which matched the volume level of peritumoral edema/invasion. Differential expression was inferred only between the “high” and “low” subgroups (52 patients). A total of 53 mRNAs (32 of which were up- and 21 down-regulated) and five microRNAs (three of which were up- and two down-regulated) were identified as differentially expressed. The detected mRNA and microRNAs were individually analyzed in ingenuity pathway analysis (IPA). As a result, the study identified, in both discovery and validation sets, PERIOSTIN (*POSTN*) as the top up-regulated gene and miR-219 as the top down-regulated microRNA in the high group. Moreover, the expression levels of *POSTN* were associated with low survival and shorter time to disease progression. These findings suggested that FLAIR could be an imaging surrogate for edema/invasion features and that POSTN inhibition could be proposed as a target for two major causes of GBM aggressiveness and failure of therapy: mesenchymal transition and cancer cell invasion. However, two limitations were present in the study: lack of image-sample registration (gene expression profiles could not be matched to a specific location on MRI) and false-positive gene hits due to a large number of probes of microarray technology. The same research group, using the same cohort of 78 patients (and 64 patients from both the TCGA and Rembrandt databases [11] as validation set), developed a non-invasive GBM classification method [12] based on lesion volume, age, and Karnofsky performance status (KPS). Using these variables, a 3-point scoring system was used to separate the patients into two VAK (volume, age, and KPS) groups: VAK-A and VAK-B. The methylation status of the O6-methylguanine-DNA-methyltransferase (*MGMT*) promoter was included in the VAK score to get the VAKM scores. VAKM patients were classified into methylated (VAK + M) and non-methylated (VAK − M). Furthermore, a total of 13,628 genes and 555 microRNAs were analyzed for significance and differential fold regulation in VAK-A and VAK-B groups. A survival benefit for VAK-A was correlated with p53 activation and with a positive *MGMT* promoter methylation status. Additionally, a total of 17 genes and eight microRNAs were significantly associated with the groups and predicted survival in an independent validation set. The findings of this study suggested that VAK classification and their molecular associations could be a robust prognostic tool and clinical trial selection criteria. However, a prospective validation was needed to confirm these results. The variance of the radial distance signal of the enhancement region of interest (ROI) was the feature most highly anti-correlated with overall survival investigated by Gevaert et al. [13] in a study performed on 55 patients. Also, 77 significant correlations between quantitative features and the VASARI (Visually AcceSAble Rembrandt Images) feature set was found. On the molecular side, four quantitative image features were correlated with molecular subtypes defined by TCGA on the basis of gene expression analysis [14]. In detail, the “mesenchymal subtype” was correlated with the minimum intensity, whereas the “classic subtype” was correlated with one necrosis and two edema image features. Another important aspect of this study was the building of a radiogenomic map based on co-expressed gene expression modules of 426 TCGA patients. The expression of a specific module—whose regulators were *GAP43* and *WWTR1* genes—had been reported to correlate with the necrotic process and with the presence of blurry edge necrotic portion of the tumor. The study demonstrated that building radiogenomic maps with quantitative imaging features and the Amaretto tool could be a promising complementary strategy toward noninvasive management of GBM. The most important limitation of this study was the manually annotated ROIs that could introduce potential observer variability. Colen et al. [15] tried to identify genomic features associated with a highly aggressive and invasive GBM imaging-phenotype using 92 patients of TCGA. The results showed that patients with deep white matter tracts and ependymal invasion on imaging (defined Class A) had a significant decrease in overall survival, whereas, in patients with the absence of such invasive imaging features (defined Class B), happened otherwise. In this context, the oncogene MYC was predicted to be the highest activation regulator in Class A. Despite some limitations, such as the unknown location of biopsy (given tumor heterogeneity in GBM, tissue origin is needed to obtain more accurate samples), this study demonstrated, for the first time, imaging features that predicted metabolic and mitochondrial dysfunction in GBM and identified the latter as possible driver for very aggressive GBM phenotypes and resistance to therapy. Mazurowski et al. [16], following the proof of concept that tumor shape likely reflects tumor growth and the genetic status of tumor cells, assumed the associations between five specific imaging features (BEVR, BEDR1, BEDR2, MF, ASD) and six genomic subtypes. For each combination of imaging features and genomic subtype, a Fisher’s exact test on 110 patients was conducted. The results showed an association between the angular standard deviation (ASD) and the genomic subtypes. In more detail, these imaging features were associated with the IDH-1p/19q subtype, RNASeq cluster, copy number cluster, and the cluster of clusters subtype (for details on these clusters, see Brat et al. [17]).

#### 2.1.2. Relations between Imaging Features and Gene Expression Data

Jain et al. [18] identified several correlations between the expression of some genes and two perfusion imaging parameters (permeability surface area product — PS and CBV). Involved genes were both pro-angiogenic (such as *TNFRSF1A*, *HIF1A*, *KDR*, *TIE1*, *TIE2*/*TEK* — positive correlations) and antiangiogenic (such as *VASH2*, *C3*, *AMOT,* and *NF1* — inverse correlations), and their expression was evaluated by gene expression microarray analysis. This study, however, was limited by small sample size (18 patients with WHO grade IV gliomas). The identified correlation could help establish a molecular basis for PS and CBV imaging biomarkers. However, as stated by authors, this study had a very low number of patients and a lack of clearly defined pathways for angiogenesis. The following study of Jain et al. [19], focused on data of 57 patients, associated with dynamic susceptibility contrast-enhanced T2-weighted MRI perfusion and gene expression data available from TCGA. This study pointed out that molecular markers underlying the Verhaak molecular GBM classification [14] could be used in combination with hemodynamic imaging biomarkers to predict patient overall survival. Another result of this study was the association between rCBVmax and patient survival rate. Using this information, Rao et al. [20] examined the molecular correlates of rCBVmax via differential expression analysis. Median rCBVmax across the entire dataset of 50 patients was used to divide the population into two groups. The differential expression analysis on the mRNA, protein, and miRNA had identified 326 genes, 76 miRNAs, and eight proteins that were differentially expressed between these two phenotype classes. Involved miRNA (miR-29b-3p, miR495–3p, miR30c/30d, miR-26a-5p, miR296-5p, miR128-3p, miR144-3p, and miR214-3p) and genes (*PTEN*, *COL15A1*, *SPARC*, *ANPEP*, *CBFB*, *STRN*, and *TMED10*) unveiled several networks of tumorogenesis interest, such as tight junction signaling and p53 signaling. Moreover, an integrated analysis revealed that the indicated miRNA/mRNA was part of a network in which miRNA directly targeted mRNAs. The results of this study indicated rCBVmax as a useful prognostic imaging biomarker. However, there were several limitations to address, such as data acquired with varying protocols from different MRI systems and the lower sample that makes essential a validation study. Molecular background of cerebral blood volume (CBV) and vessel size (VS) of capillaries in GBM was instead studied by Heiland et al. [21] on 21 patients from a private collection (484 samples from the GBM cohort were used as validation cohort). For this purpose, transcriptional data was analyzed by weighted gene co-expression network analysis (WGCNA). Ten modules were highly correlated to CBV and VS. *ARAF/TRAF* was identified as hub-genes of the highest CBV correlating module. One module was exclusively associated with CBV, showing enrichments in the epithelial growth factor (EGF). Moreover, patients with increased CBV and VS mainly showed a mesenchymal gene expression. Also, in this case, the main limitation was the small number of cases in the discovery set. Moreover, replication of the analyses on the TCGA data corroborated the results. Verhaak gene expression classification was also used by Gutman et al. [22], but, in this case, using 75 cases, associations were found with contrast-enhanced tumor characteristics. Up-regulation of oncogene MYC was also identified, in a later study on 99 patients of the same group [23], as the upstream regulator of female GBM cell death phenotypes. Instead, *TP53* played a different role in male-female, it was significantly up-regulated in the high cell death of male patients, and down-regulated in female patients. The novelty of this study was the identification of sex-specific molecular mechanisms for cell death in patients with GBM. A clustering analysis, executed on 92 cases by Rao et al. [24], revealed that a combination of three features (volume-class, hemorrhage, and T1/FLAIR-envelope ratio) significantly stratified survival in two phenotypes. In more detail, a low value for any of these three features showed favorable survival. Moreover, differential expression analysis suggested that some immune-associated and metabolism-associated pathways affected the transition between the two phenotypes. Integrating molecular data suggested the roles of several genes/miRNA regulating proliferation (*PGC1alpha*/miR-199a, miR-125, and miR-129) and invasion (*PAR1*, *HOXC6* / miR-499, miR-146b). The authors of this study suggested that combinatorial radiophenotype could be used as a screening tool for assessing response to drugs that target invasion features and glioma cell proliferation. A pathway recognition algorithm using data integration on genomic models (PARADIGM) [25] was used by Itakura et al. [26] to integrate gene expression and copy number variation data of 144 patients in order to associate these features with three different image clusters. Up-regulation of the c-Kit stem cell factor receptor pathway was found to be correlated with pre-multifocal cluster 1, while down-regulation of 21 pathways, including c-Kit, *VEGFR* signaling, *PDGFR-α* signaling, *FOXA* transcriptional networks, and angiopoietin (*Ang*)/*Tie2,* was found to be correlated with spherical cluster 2. Finally, the up-regulation of 31 pathways, including canonical *WNT* and *PDGFR-β* signaling, and the down-regulation of many of the vascular pathways in cluster 2, such as *VEGFR* and *Ang/Tie2*, were found to be correlated with the rim-enhancing cluster 3. In summary, the author proposed possibilities for target identification and unique therapeutic strategies for each subtype discovered. Qian et al. [27] used a multi-task longitudinal sparse regression method to show the associations between 119 genes and 225 morphological features extracted from the longitudinal MRI of 38 patients (of them, 21 were from TCGA/TCIA database and were used as validation set). The analysis indicated a significantly higher expression level of *IRF9* and *XRCC1* in PsP (diagnosis for pseudoprogression) cases than those for the true tumor progression (TTP) patients in both private and TCGA/TCIA data. The novelty of this study was based on the introduction of a new longitudinal sparse regression model to construct the relationship between imaging features and gene expressions. TCGA-TCIA validation cohort confirmed the authors’ findings; however, the main limitation was the low sample size of the discovery cohort. Liu et al. [28] used a gene set enrichment analysis (GSEA) to identify up-regulated gene sets and pathways that were different between two PWI (Perfusion weighted)-based clusters. The cluster II, which was defined by elevated PWI features, was characterized by enrichment of angiogenesis and hypoxia pathways. The genes involved in both the hypoxia signaling and the angiogenesis pathways consisted of angiogenin (*ANG*), *VEGF-A*, and transforming growth factor-beta 2 (*TGFB2*). The study involved 48 patients from TCGA (69 patients from Stanford University Medical Center were used as validation set) and provided the first approach leveraging PWI features as potential imaging biomarkers to classify patients for personalized antiangiogenic treatment. However, several limitations were present: a small number of patients with complete treatment information and “batch effects” between the two cohorts due to variability in perfusion imaging technique and variability in antiangiogenic treatments administered. Using 84 patients’ TCGA data as validation cohort (and Chinese Glioma Genome Atlas—CGGA as training set), the same group [29] have performed a radiogenomic analysis showing the ability of radiomic features to predict progression-free survival and their association with the immune response, programmed cell death, cell proliferation, and vasculature development, as reported by transcriptomic data. Liao et al. [30], using a machine learning approach on data from 46 patients, showed high or moderate correlations between differential expression of three patterns of gene expression—also defined metagenes (*TIMP1*, *ROS1*, *EREG*) and image features. In particular, *EREG* was found positively associated with dependence non-uniformity, difference average, contrast, and cluster prominence. Inverse difference zone variance, large area emphasis, and root mean squared were found to be negatively associated. *ROS1* was found to be negatively associated with inverse difference moment and, finally, *TIMP1* was found to be positively associated with contrast and cluster prominence and negatively associated with inverse difference, zone variance, and large area emphasis. Overall findings indicated that association of genes or microRNAs with the imaging features (listed above) might enable researchers to screen for molecular cancer subtypes and biological mechanisms (angiogenesis and cellular invasion) involved in tumor aggressiveness. However, for future analysis, the larger patient sample size is needed to better assess the predictive model found and validate experimentally the candidate genes/miRNAs. In concerns with FLAIR data, additional sequences and imaging modalities can be combined for multi-modal analysis, which can provide comparison results about different methods. Furthermore, for imaging feature selection, an advanced dimensionality reduction method could improve dimensionality reduction and classification performances.

#### 2.1.3. Relations between Imaging Features and Mutation Data

The aforementioned study of Gutman et al. [22] also showed that there were weak correlations between imaging features and copy number variation, in particular, a weak association between *CDKN2A* deletion and necrosis, amplification of *EGFR,* and an increased percentage of contrast enhancement. Correlations between 11 MRI-derived volumetric features and mutation status have been also studied by Gutman et al. [31] in a data set of 76 patients. Some significative mutations were related to volumetric features: *EGFR* mutations displayed a significantly higher necrosis/contrast enhancing ratio and a significantly lower contrast-enhancing/tumor bulk ratio. Furthermore, *RB1* mutations tumors showed significantly smaller T2-FLAIR hyperintensity, and *TP53* significantly predicted necrosis and tumor volumes by contrast-enhancing. Additionally, NF1 mutation status was significantly predicted by contrast-enhancing volume and tumor bulk volume, whereas *PDGFR-α* was significantly predicted by T2-FLAIR hyperintensity/total tumor volume and tumor bulk/total tumor volume ratios. These results could impact personalized medicine and propose imaging features as a predictor of genetic variants useful as a noninvasive technique in clinical practice and the choice of the treatment. The main limitation of the study, as in the other cases, was the unknown exact location of the biopsy. Nicolasjilwan et al. [32], through multivariate Cox’s models, analyzed associations between combined biomarkers (clinical data, VASARI imaging features, and genomic variations) and overall survival. AUC (area under the curve) analysis of different combinations of biomarkers led to the conclusion that the model, including all three types of data, was the best predictor for survival. The features that were significantly associated with survival were the proportion of tumor contrast enhancement on MRI and *HRAS* copy number variation. Jain et al. [18] tried to correlate only KPS with four Verhaak molecular subclasses of GBM and survival, but no association was found. In the same study, which included 45 patients, combining epidermal growth factor receptor (*EGFR*) alteration (mutation or amplification) with a relative cerebral blood volume of the non-enhancing region (rCBVNER), a significant association with OS (overall survival) was found, with worst survival in the high-rCBVNER wild-type *EGFR* group. In summary, perfusion parameter rCBVNER provided important prognostic information that could be complementary to clinical and genomic features, and this association could help to refine the prognostic models in patients with GBM. The prognostic value of these findings, however, limited by some not well-represented features and by the limited population used.

#### 2.1.4. Relations between Imaging Features and Proteomic Data

To search for a relationship between tumor MRI features and proteomics data, Lehrer et al. [33] conducted a “radio-proteomic” analysis of LGG on 57 patients. The combination of imaging and protein abundance data showed VASARI imaging features significantly associated with several changes in protein level. The strongest associations found were: T1/FLAIR ratio with down-regulation of *AMPK* and acute myeloid leukemia signaling; MRI necrosis with up-regulation of *PI3K/AKT/mTOR* signaling and apoptosis, although correlated with down-regulation of *AMPK* and protein kinase A signaling; edema with increased *NGF* signaling and G1/S checkpoint regulation; the presence of cysts with decreased *PI3K/AKT* and phospholipase C signaling; localization of tumor to the parietal lobe with up-regulated p53 signaling activity and with down-regulated *IL-8* signaling. The authors hypothesized that imaging and protein abundance data might reveal imaging biomarkers tying radiological features to the proteomics of LGG.

### 2.2. Breast Cancer (BRCA)

**Figure 2:**
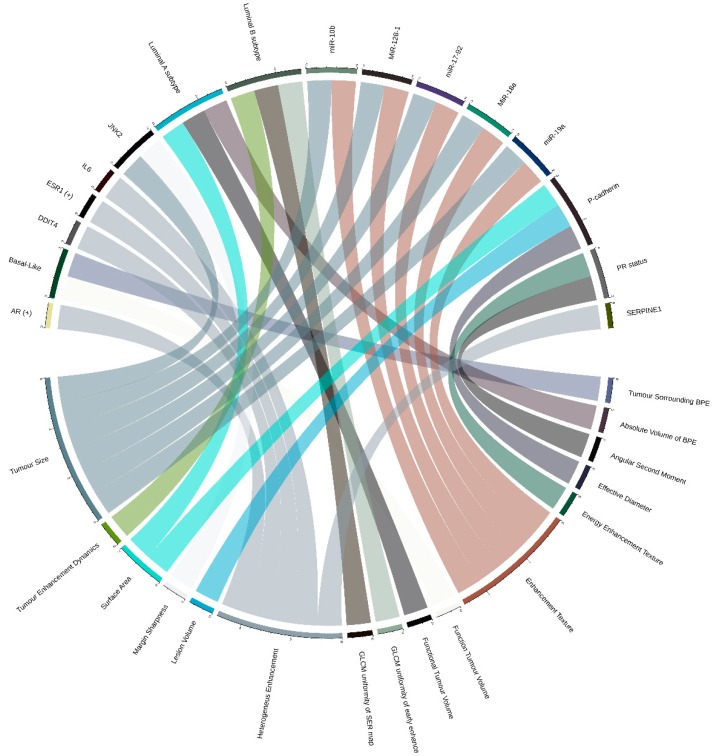
Radiogenomic associations in TCGA-TCIA BRCA (Breast Cancer). Molecular omic features are represented on the top of the image, while imaging features are represented on the bottom. The arcs represent relations, and (+) indicates a positive relation.

The majority of radiogenomic literature on breast cancer [34] relies almost exclusively on DCE-MRI (dynamic contrast-enhanced MRI) imaging features and, in some cases, on positron emission tomography/magnetic resonance (PET/MR) imaging [35,36]. Ten TCGA-TCIA-based studies were included in our review, and most of them aimed to capture associations between imaging features and breast cancer intrinsic molecular subtypes. The more detailed relations by the original articles are graphically represented in Figure 2.

#### 2.2.1. Relations between Imaging Features and Cancer Subtypes

The first study [37] investigated relations between the intrinsic subtypes and semi-automatically extracted MRI features in 48 patients from four different institutions. Only one relation was identified between the imaging feature F1, linked to tumor enhancement dynamics, and the luminal B subtype. A common limitation characterized this study: the limited sample size representative for each subtype, i.e., only eight patients in the luminal B subtype. However, the ability to identify breast cancer molecular subtype without an invasive genetic analysis has prominent clinical benefits. Wu et al. [38], using 84 patients from private institution and 126 patients from TCGA as a validation test, extracted 35 DCE-MRI quantitative image features (including morphology, texture, and volumetric features) and built sparse logistic regression models to identify three intrinsic subtypes (luminal A, luminal B, and basal). Six features enabled distinction across intrinsic subtypes. In particular, surface area, functional tumor volume, and absolute volume of BPE (breast parenchymal enhancement) distinguished luminal A and non-luminal A patients, whereas GLCM (gray-level-co-occurrence matrix) uniformity of early enhancement map and GLCM uniformity of SER (signal enhancement ratio) map distinguished luminal B and non-luminal B patients. Function tumor volume and tumor surrounding BPE fraction were able to distinguish basal-like and non-basal-like patients. The ability to accurately identify breast cancer molecular subtypes has important therapeutic implications, but the results of this study needed to be further validated in larger prospective cohorts. Prediction analysis of the molecular classification of breast cancer was instead performed by Li et al. [39] on a dataset of 91 patients. The Li et al. [40] group also investigated the relationship between MRI features and multigene assays of MammaPrint [41], Oncotype DX [42], and PAM50 [43]. The study showed significant associations between radiomics features, such as tumor size and enhancement texture (indicators of tumor heterogeneity), and multigene assay recurrence scores. New breast cancer imaging subtypes were instead identified by Wu et al. [44] through unsupervised consensus clustering of quantitative image phenotypes of the tumor and background parenchymas. The three distinct imaging subtypes identified (homogeneous intratumoral enhancing, minimal parenchymal enhancing, and prominent parenchymal enhancing) were subsequently related to the expression of 692 genes through a gene expression-based classifier.

#### 2.2.2. Relations between Imaging Features and Gene Expression Data

Unsupervised hierarchical cluster analysis was used by Kim et al. [45] to group 70 patients in two major subgroups based on different microarray gene expressions. According to the breast imaging reporting and data system (BI-RADS) MRI lexicons of mass [46], a data system to standardize disease risk stratification criteria for non-radiologist, internal enhancement was found significantly different between two groups. In particular, heterogeneous enhancement was found most frequently in group 1, while rim enhancement was found dominantly in group 2. Moreover, the authors found 1303 genes significantly associated with each group. In particular, group 1 showed a significantly higher expression of *AR* and *ESR1*. The study had some limitations, such as small sample size and unavailable information on the long-term outcome to relate prognosis and MRI features or specific genes enriched in each group. Using data from 87 patients, Fan et al. [47] identified a link between prognostic imaging features (skewness, correlation, and maximum probability in S-0, and kurtosis, skewness, median value, and maximum probability) from DCE-MRI data and gene expression modules. Wu et al. [48], using data of the 126 samples with dynamic contrast-enhanced MR imaging, found an association of heterogeneous enhancement patterns of tumor-adjacent parenchyma and the tumor necrosis signaling pathway with tumor gene expression data. In particular, some of the involved genes were *IL6*, *SERPINE1,* and *DDIT4*. These results confirmed the enhancement pattern of breast parenchyma as a promising imaging marker for the risk of developing breast cancer. Moreover, the importance of the enhancement pattern study was also due to its role in breast cancer treatment response, local recurrence, and survival.

#### 2.2.3. Relations between Imaging Features and Multiple Molecular ‘Omic Features

Guo et al. [49] used radiogenomic features to predict clinical outcomes of the same 91 patients used by Li et al. with a study design based on DCE-MRI, clinical, and genomic (copy number, gene expression, and DNA methylation) data. All tumor size features were found significantly positively associated with tumor stage, while irregularity feature was found to be significantly positively related to tumor stage. One tumor margin feature (variance of radial gradient histogram) and two enhancement texture features (inverse difference moment and sum average) were also predictive of tumor stage. They found PR (Progesteron Receptor) status in association with the angular second moment—energy enhancement texture. Interestingly, some clinical-pathological characteristics were mainly associated with radiomic features (tumor stage), and others (such as copy number alteration of *CDK4* and *PTEN2* and gene expression of *BCL2* for ER —Estrogen receptor) performed better than radiomic features in predicting hormone receptor status (ER/PR). Another interesting result of this study was the non-improvement of the prediction of clinical outcomes performance by combining genomics and radiomic features. Despite this study to date the largest study that combines multiple types of genomic data with the radiomic one in predicting breast cancer prognosis, the authors stated as major radiogenomic limitation the small sample size of 91 cancer cases with 38 radiomic features and 144 genomic features. On the same dataset of 91 patients, Zhu et al. [50] found several quantitative MRI features (such as tumor size, shape, margin, and blood flow kinetics) associated with different molecular profiles, such as DNA mutation, miRNA expression, protein expression, pathway gene expression, and copy number variation. In particular, a total of 1103 statistically significant pathways and radiomic phenotypes relationships were reported. Statistically significant associations were also found between miRNA expressions (mir-128-1, mir-18a, mir-19a, mir-17-92, mir10b) and two radiomic features: tumor size and enhancement texture. Moreover, interesting associations between protein levels (P-cadherin and *JNK2*)/radiomic phenotypes (effective diameter, surface area, and lesion volume for P-cadherin and tumor size and margin sharpness for *JNK2*) and somatic gene mutations/radiomic phenotypes were also found.

### 2.3. Clear Cell Renal Cell Carcinoma (KIRC)

Renal cell cancer is the most frequent kidney cancer disease, classified into clear cell renal cell carcinoma (ccRCC) and papillary renal cell carcinoma (pRCC), with corresponding datasets available in TCGA/TCIA. ccRCC is the most common subtype of renal cell carcinoma (RCC), and it is distinctly caused by a somatic mutation in the Von Hippel-Lindau (*VHL*) tumor suppressor gene, even if several other genes have been associated with the advanced form of the disease using whole-genome sequencing. Only four studies based on TCGA-TCIA data were included in our review, and two of these could not be described as real radiogenomics study (for a complete review on radiogenomics in RCC, see Alessandrino et al. [51]). The first study [52], conducted on MRI and computed tomography (CT) data of 103 patients, investigated associations between imaging features and the mutational status of ccRCC. In detail, mutations in two distinct genes were associated with radiomic features: in the *BAP1* gene, associated with ill-defined tumor margins and with the presence of calcification, and in the *MUC4* gene, associated with exophytic growth. *BAP1* mutation status was also predicted by Ghosh et al. [53], who developed an imaging-genomic pipeline able to obtain 3D intra-tumor heterogeneity features (textural, volumetric, and ratio features) from contrast-enhanced CT images and associated them with gene mutation status. Seventy-eight patients with diagnostic pretreatment CT scan from TCIA were used as a dataset, but, since the pipeline was able to detect a single mutation status, the study was not classifiable as radiogenomic. Moreover, there was an important limitation: none of the associations were significant after adjusting for multiple testing. The authors explained this result due to the small number of patients available for each gene mutation. Also, Kocac and colleagues reported a study based on one-gene prediction [54]. Their aim was to evaluate machine learning (ML)-based high-dimensional quantitative CT texture analysis in predicting the mutation status of the gene Polybromo 1 (PBRM1). Using 10 selected features, the artificial neural network (ANN) algorithm accurately classified 88.2% (142 of 161 patients used as dataset - AUC value, 0.987). Using five selected features, the random forest (RF) algorithm correctly classified 95% (153 of 161 patients—AUC value, 0.987). Gene expression profiling was another way used to investigate radiogenomic associations in ccRCC. In particular, Bowen et al. [55], analyzing 177 patient data, showed associations between CT imaging features and ccRCC subtypes (m1–m4) defined by different mRNA expression profiles [56]. m1 tumors (defined by genes related to chromatin remodeling processes and a higher frequency of *PBRM1* gene mutations) were found positively correlated with a well-defined margin. On the other hand, m3 tumors (defined by frequently deletion of *CDKN2A* and mutations in *PTEN*) were found negatively correlated with CT features that are considered indicators of the infiltrative/invasive phenotype (well-defined margin, renal vein invasion, and urinary collecting system invasion). Also, in this study, there were several limitations: image data sets were extremely heterogeneous (different scanner modalities, manufacturers, and acquisition protocols were used), and, in most cases, the images were not acquired as part of a controlled research study or clinical trial. Moreover, several tumor features were more often represented in certain subgroups.

### 2.4. Other Tumors

Among radiogenomics studies using TCGA/TCIA data, two papers did not fit into any three tumors described above. One of these reported high-grade serous ovarian cancer. In this work, Vargas et al. [57], using 92 patients, found an association of CT imaging features with both time-to-disease progression (TTP) and the transcriptomic profile defined CLOVAR (classification of ovarian cancer), which included four subtypes: differentiated, immunoreactive, mesenchymal, and proliferative [58]. Two CT features, presence of peritoneal disease in the pouch of Douglas and higher number peritoneal disease sites, were found to be associated with mesenchymal subtype. The second paper discussed the study of the chromosomal instability (CIN) of gastric cancer. About half of all gastric cancers are CIN subtypes characterized by the high rate of gain or loss of whole chromosomes. In this work, Lai et al. [59], using 40 patients, investigated the role of computed tomography (CT) imaging features in predicting the CIN status of gastric cancer. From the training set, two CT imaging features enabled prediction of contrast-induced nephropathy (CIN) status of gastric cancer: smaller tumor diameter and acute tumor transition angle. The CIN status of tumors could be detected by cytogenetic techniques, such as comparative genomic hybridization or single nucleotide polymorphism array-based methods. These techniques might not provide information for decision-making in cancer treatment. Moreover, the tumor sample is not always available, and then the clinical impact of using imaging data for an earlier and more precise diagnosis of CIN subtype might provide complementary information in the absence of genomic profiles.

## 3. Discussion

Overall papers described here, carried out a radiogenomics study based on the evaluation of possible associations or correlations between imaging and genomic features that could reflect a disease phenotype-genotype relationship (Table 2 summarizes the most important relations) for more precise diagnosis, classification of intra-tumor heterogeneity, and prediction of clinical outcomes. So far, glioblastoma and breast cancer are the most represented tumor in radiogenomics works based on TCGA-TCIA (Table 1). From the radiomics point of view, imaging biomarker candidates need to own some features like independence and reproducibility to be a robust biomarker. Indeed, radiomic features are often influenced by relevant parameters, among which the most important is image pre-processing and data acquisition. In this scenario, operations, such as reproducibility and initiatives, that promote technical standardization of patient’s scanned images, are becoming fundamental to provide guidelines and standardized procedures. From the selected literature, it seems clear that there are several common limitations in the selected radiogenomic studies. The most frequent are:

1. lack of image-sample registration (gene expression profiles cannot be matched to a specific location on MRI),
2. manually annotated ROIs that could introduce potential interobserver variability,
3. unknown location of biopsy (given tumor heterogeneity, tissue origin is needed to obtain more accurate samples),
4. data acquired with varying protocols from different MRI systems. Image data can be extremely heterogeneous due to different scanner modalities, manufacturers, and acquisition protocols,
5. in most cases, data were not acquired as part of a research protocol or clinical trial.

Moreover, the identification of radiogenomic biomarkers requires a large amount of different data, especially with the recent adoption of machine learning and deep learning techniques in the data analysis field. Indeed, published radiogenomic papers, summarized in several radiogenomic reviews [3,60,61], are often based on a limited sample size, especially when the study is performed on a private collection. In addition, a common scenario consists of the availability of imaging data to be associated with a large amount of genomic data. This creates a problem that is often addressed through the creation of gene modules, even though this might weaken the potential of outcome predictions. Another limitation arising from radiogenomic data is that the number of imaging or molecular ‘omics features can be significantly larger than the number of available patients for the study. This issue, defined as “curse of dimensionality”, leads to non-generalizable results and overfitting. To reduce this, advanced algorithms able to order features by their significance for a given outcome are often crucial. Moreover, the genomic analyses are usually performed on a sample that hardly reflects the tumor heterogeneity, which is well represented by imaging data. This can create an inconsistency between the two types of data. In addition, the radiogenomic relations may be also dependent on the patient, clinical, and environmental data, which are not always available. Most of these issues and limitations can be overcome through interdisciplinary collaboration, standardization of data and methods, use of standard data structures [62], and the availability of public multidimensional datasets. Indeed, since a large amount of data cannot be found within a single research group or institution, currently, retrospective datasets assembled from different institutions have become a necessity. In this scenario, a leading role is played by TCGA and TCIA, which contains molecular omic data of 67 different tumor primary sites and, in some cases, corresponding public imaging datasets available through TCIA archive. However, in most cases, available data on TCIA are only raw images and metadata, while, only for some projects, also extracted features are available in the “Analysis results directory” section of the TCIA website. This creates a discrepancy compared with ‘omic data of TCGA, where most of the projects contain raw data and several processed data. This issue added to the well-known of inter- and intra-institutional data heterogeneity is the main reason for the small patient population found in several studies, upon inclusion criteria of radiogenomics workflow. In this scenario, a direct link between TCGA and TCIA data type availability, that allows knowing in advance if a radiogenomics study is feasible using available data, becomes essential. Even if there is a need of advanced tools to better link imaging and molecular omic data, the combination of TCGA-TCIA data is a unique and extensive publicly available collection of cancer data, providing researchers with a great opportunity to increase tumor understanding and to better set up a study using available data as validation set of a hypothesis.

**Table 2:** Imaging—molecular omic feature associations. (–) indicates a negative relation, (+) a positive relation, (m) mutation of the corresponding gene, (l) a low value of the corresponding feature, and (h) a high value.

Paper	Imaging Features	Molecular Omic Features	TCGA/TCIA Number of Patients	Internal Cohort/ n° Patients	Statistical Analysis
**Glioblastoma Multiforme (GBM) and Low Grade Glioma (LGG)**					
[10]	FLAIR signal volume	POSTN (+), miR-219 (–)	78 (39 of which as validation set)	No	Comparative marker selection (CMS)
[12]	Lesion Volume (+ age and KPS)	P53 activation, MGMT methylation	78 (+ 64 from TCGA and Rembrandt)	No	Cox proportional hazards likelihood ratio
[18]	rCBVner (h)	Wild-type EGFR	45	No	Analysis of variance
[19]	PS, CBV	TNFRSF1A, HIF1A, KDR, TIE1, TIE2/TEK (+), VASH2, C3, AMOT, and NF1 (–)	18	No	Pearson correlation coefficient
[20]	rCBVmax	miR-29b-3p, miR495-3p, miR30c/30d, miR-26a-5p, miR296-5p, miR128-3p, miR144-3p and miR214-3p, PTEN, COL15A1, SPARC, ANPEP, CBFB, STRN, TMED10	50	No	Two-sided t-test
[21]	CBV	EGF pathway (ARAF/TRAF)	484 (validation set)	Yes/ 21 (discovery cohort)	Cox-regression tests
	VS	HIF1A, BNIP3L			
[13]	Blurry edge necrotic portion	GAP43 (–), WWTR1 (–)	426	No	Amaretto modules
[15]	Deep white matter tracts, ependymal invasion	myc (+)	92	No	Comparative marker selection (CMS)
[23]	Volumes of necrosis	myc (+ in female), TP53 (– in male)	99	Yes/ 369 (validation set)	Comparative marker selection (CMS)
[24]	Volume-class, hemorrhage, T1/FLAIR-envelope ratio	PGC1alpha, PAR1, HOXC6, miR-199a, miR-125 and miR-129, miR-499, miR-146b	92 (48 of which as validation set)	No	Comparative marker selection (CMS)
[31]	Necrosis/contrast enhancing ratio (h), Contrast-enhancing/tumor bulk ratio (l)	EGFR (m)	76	No	Two-sided student’s t test
	T2-FLAIR hyperintensity	RB1 (m)			
	Necrosis/contrast enhancing volume	TP53 (m)			
	Contrast enhancing volume, Tumor bulk volume	NF1 (m)			
	T2-FLAIR hyperintensity, Total tumor volume, Tumor bulk/total tumor volume ratio	PDGFRA (m)			
[26]	Pre-multifocal cluster	c-Kit (+)	144 (validation set)	Yes/121 (discovery cohort)	SAM (FDR < 15% for imaging features and FDR < 5% for signaling pathways)
	Spherical cluster	VEGFR (–), PDGFR (–), FOXA (–), Ang/Tie2 (–)			
	Rim-enhancing cluster	WNT, PDGFR-β, VEGFR, Ang/Tie2			
[27]	PsP	IRF9 (+), XRCC1 (+)	21 (validation set)	Yes/17 (discovery cohort)	Multi-task longitudinal sparse regression
[28]	PWI features (h)	ANG, VEGF-A, TGFB2	48	Yes/79 (validation set)	Random forest model
[16]	ASD	IDH-1p/19q (m)	110		
[33]	T1/FLAIR ratio	AMPK (–)	57	No	Agglomerative unsupervised hierarchical clustering (FDR < 0.25)
	Necrosis	PI3K/AKT/mTOR (+), AMPK (–), PKA (–)			
	Edema	NGF (+), GS1 signalling (+)			
[30]	Dependence non-uniformity, Difference average, Contrast and cluster prominence	EREG (+)	46	No	Pearson correlation analysis
	Inverse difference zone variance, Large area emphasis, Root mean squared	EREG (–)			
	Inverse difference moment	ROS1 (–)			
	Contrast, Cluster prominence	TIMP1 (+)			
	Inverse difference moment, Zone variance, Large area emphasis	TIMP1 (–)			
**Breast Cancer (BRCA)**					
[37]	Tumor enhancement dynamics	Luminal B subtype	48	No	Multivariate logistic regression models (associations FDR < 0.0022)
[38]	Surface area, Functional tumor volume, Absolute volume of BPE	Luminal A subtype	126 (validation set)	Yes/ 84 (discovery cohort)	Multivariate logistic regression models (associations FDR < 0.25)
	GLCM uniformity of SER map, GLCM uniformity of early enhancement map	Luminal B subtype			
	Function tumor volume, Tumor surrounding BPE	Basal-Like			
[49]	Angular second moment, Energy enhancement texture	PR status	91	No	Logistic regression and *t*-test (associations FDR < 0.1)
[50]	Tumor size, Enhancement texture	MiR-128-1, MiR-18a, miR-19a, miR-17-92, miR-10b	91	No	Regression analysis and clustering analysis (FDR ≤ 0.05)
	Effective diameter, Surface area, Lesion volume	P-cadherin			
	Tumor size, Margin sharpness	JNK2			
[45]	Heterogeneus enhancement	AR (+), ESR1 (+)	70	No	Unsupervised hierarchical cluster and *t*-test (no multiple hypothesis testing)
[48]	Heterogeneus enhancement	IL6, SERPINE1, DDIT4	126	No/ 879 (Independent cohort) + 159 (Independent cohort, GEO data set)	Univariate analysis (associations FDR < 0.1)
**Clear Cell Renal Cell Carcinoma (ccRCC)**					
[52]	Ill-defined tumor margins	BAP1	103	No	Pearson’s χ2 test and the Mann–Whitney U test (no significant associations after adjusting for multiple hypothesis testing)
	Exophytic growth	MUC4			
[55]	Well-defined margin	PBRM1 (m) (+)	177	No	Multivariate logistic regression analysis
	Well-defined margin, Renal vein invasion, Urinary collecting system invasion	CDKN2A (m), PTEN (m)			
**Others**					
[57]	Presence of peritoneal disease in the pouch of Douglas, Higher number peritoneal disease sites	The mesenchymal subtype of high-grade serous ovarian cancer	92	No	Multivariate logistic regression analysis (associations FDR < 0.1)
[59]	Smaller tumor diameter and acute tumor transition angle	Contrast-induced nephropathy (CIN) status	40	Not specified/18 validation cohort	Multivariate logistic regression analysis (no multiple hypothesis testing)

FLAIR: Fluid Attenuated Inversion Recovery, POSTN: Periostin, MGMT: O6-methylguanine-DNA methyltransferase, TCGA: The Cancer Genome Atlas, rCBVner: Relative Cerebral Blood Volume of NER, EGFR: Epidermal growth factor receptor, PS: Permeability Surface, CBV, Cerebral Blood Volume, TNFRSF1A: TNF receptor superfamily member 1A, HIF1A: Hypoxia Inducible Factor 1 Subunit Alpha, KDR: Kinase Insert Domain Receptor, TIE1: Tyrosine Kinase With Immunoglobulin Like And EGF Like Domains 1, TEK: TEK Receptor Tyrosine Kinase, VASH2: Vasohibin 2, C3: Complement 3, AMOT: Angiomotin, NF1: Neurofibromin 1, TEN: Phosphatase and tensin homolog, COL15A1: Collagen alpha-1(XV) chain, SPARC: Secreted protein acidic and rich in cysteine, ANPEP: Alanyl aminopeptidase, CBFB: Core-binding factor subunit beta, STRN: Striatin, TMED10: Transmembrane P24 trafficking protein 10, EGF: Epithelial growth factor, VS: Vessel Size, BCL2: Interacting Protein 3 Like, GAP43: Growth associated protein 43, WWTR1: WW domain-containing transcription regulator 1, TP53: Tumor protein P53, PGC1alpha: Peroxisome proliferator-activated receptor gamma coactivator 1-alpha, PAR1: Prader Willi/Angelman region RNA 1,HOXC6: Homeobox C6, RB1: Retinoblastoma protein, PDGFRA: Platelet-derived growth factor receptor A, VEGFR: Vascular endothelial growth factor receptor, Ang: Angiopoietin, Tie2: Tyrosine-protein kinase receptor Tie-2, WNT: Wnt family member 1, FOXA: Forkhead Box A1, IRF9: Interferon regulatory factor 9, XRCC1: X-ray cross-complementing, PsP: Pseudoprogression, PWI: Perfusion weighted imaging, ANG: Angiogenin, TGFB2: Transforming growth factor-beta 2, ASD: Angular standard deviation, AMKP: AMP-activated protein kinase, PI3k: Phosphoinositide 3-kinase, AKT: Serine/threonine kinase 1, mTOR: Mechanistic target of rapamycin kinase, NGF: Nerve growth factor; IDH: Isocitrate Dehydrogenase (NADP(+)) 1, ROS1: ROS proto-oncogene 1, EREG: Epiregulin, TIMP1: TIMP metallopeptidase inhibitor 1, AR: Androgen receptor, ESR1: Estrogen Receptor 1, IL6: Interleukin 6, DDIT4: DNA damage-inducible transcript 4, BAP1: BRCA1 associated protein 1, MUC4: Mucin 4 Cell surface associated, PBRM: Polybromo-1, CDKN2A: Cyclin Dependent Kinase Inhibitor 2A, PTEN: Phosphatase and tensin homolog.

## 4. Methods

### 4.1. Search Strategy and Articles Selection

We systematically searched the biomedical literature through PubMed and the ISI Web of Knowledge for papers published until 30-10-2019 on the radiogenomics field. The search was based on the following words: “The Cancer Genome Atlas and radiogenomics”, “The Cancer Imaging Archive and radiogenomics”, “TCGA TCIA radiogenomics”. In addition, we checked existent publications and reviews on radiogenomics on the TCGA-TCIA website section for other available studies. The articles were classified according to the tumor site, and, for each of them, we described the size of the used dataset and the main results of the radiogenomic aspect of the study. As mentioned, many studies referred to “radiogenomic”, often were not conceived into an overall omic framework, which is a proper radiogenomics. Unequivocal radiogenomic studies included in this review were manually-assessed by two people in order to select publications that fulfill the following inclusion criteria: (1) full-text available in English; (2) the article comprised statistically assessed associations between imaging features and molecular omic data; (3) both TCGA and TCIA data were used for each study. These papers are summarized in Table S1, according to the tumor type, and their main findings are discussed in the corresponding result subsection.

### 4.2. Graphic Representation of Feature Associations

All figures were built through the Circlize R package [63]. The images represented the association between imaging features (radiomic and/or radiological) and molecular ‘omic signature, which could be gene expressions, mutations, clusters, or cancer subtypes. Only the more detailed associations, reported in the original articles, are graphically represented. The associations not represented in the images are shown in Table 2.

## 5. Conclusions

TCGA-TCIA represents the largest data repository that offers clinical, imaging, and molecular omic data for the same set of patients, making them an extremely important resource to perform radiogenomic analysis. The selected literature showed that these data were used as testing data and/or validation set [14,25,39] (in some cases, the validation data is a subset of the testing set) or also as a set to test a pipeline [42]. Of the total number of 35 selected articles, 21 analyzed GBM data, 10 analyzed BRCA data, and two analyzed ccRCC data. Radiogenomic frameworks of the selected studies were based on hierarchical clustering or multiple hypotheses testing (explorative approach), where a lot of imaging features were tested against several molecular omic features. Other studies described here, on the contrary, were hypothesis-driven-based. In this case, a specific relationship was tested. Our systematic review highlighted that, to date, the main advances in radiogenomics cover mainly brain tumors, such as glioblastoma and breast cancer. Major imaging biomarkers that emerged for brain tumors, especially due to more validated segmentation methods, allow better discrimination of lesions with respect to the other oncological studies. Among the imaging techniques, we found that functional diffusion-weighted imaging (DWI) magnetic resonance and positron emission tomography (PET) statistical features of first and higher-order enabled better characterization of tumor phenotypes. Collectively, the radiogenomics study described the sufferings of small size populations and lack of standardization, especially for radiomic workflow (imaging acquisition instrumentation, segmentation, feature extraction, and data analysis). Radiogenomic studies aimed to find imaging biomarkers correlating to tumor phenotype and genotype of the disease. Based on the described studies, we could conclude that great attention and precautions are needed for radiogenomics study design. First of all, to evaluate the statistical significance of the imaging descriptors and genomic signature, multiple hypothesis testing, such as the Benjamini-Hochberg method should always be taken into account. Another crucial point is the validation of the predictive model on an external dataset with appropriate sample size. In this concern, we found that public databases, such as TCGA-TCIA data, are an unprecedented resource of independent cohorts. Once a relationship between imaging features and molecular omic signatures is established, a major challenge in radiogenomics remains a deep insight into the biology underlying tumor phenotype. This is especially due to the lack of protocol standardization technique across multicenter studies, for both omic data generation and processing, making it difficult to replicate the experimental conditions and associations found in different studies. Moreover, another limitation using TCGA/TCIA data as a discovery/validation cohort for radiogenomic study is the lack of links between imaging and molecular ‘omics data. In recent years, many advances in radiomics and genomics have been made. For instance, deep learning methods are leading to higher accuracy of volume lesion segmentation and would have a profound impact on many applications to pursue precision medicine in the near future. For TCGA-TCIA data, handling several tools, portal, and data structures are emerging to facilitate radiogenomic studies and reduce the discrepancy of processed available data between TCGA and TCIA [62]. Ideally, quantitative descriptors of medical images might replace the ex vivo biopsy profile of the tumor. It is more likely that radiogenomics would achieve the characterization of disease phenotype through a large number of features from medical images correlating with molecular and clinical tumor characteristics. In this way, the clinical potential application of radiogenomic might be non-invasive in vivo tumor characterization to extract prognostic and predictive data, for more precise diagnosis and monitor patients’ treatment response.

## Figures and Tables

**Table 1:** Unequivocal radiogenomic studies using TCGA-TCIA data.

Tumor Type	TCGA/TCIA Project	Sum of Study
Glioblastoma and low grade glioma	GBM and LGG	21
Breast cancer	BRCA	10
Clear cell renal carcinoma	KIRC	2
Other	OV and STAD	2

GBM: Glioblastoma Multiforme, LGG: Low-Grade Glioma, BRCA: Breast cancer, KIRK: Kidney Renal Clear Cell Carcinoma, KIRC: Kidney Renal Clear Cell Carcinoma, OV: Ovarian Cancer, STAD: Stomach adenocarcinoma.

## Supplementary Materials

Supplementary materials can be found at https://www.mdpi.com/1422-0067/20/23/6033/s1. Table S1. Radiogenomic studies with TCGA/TCIA data.

## Abbreviations

NIMH	National Institute of Mental Health
NDA	National Institute of Mental Health Data Archive
TCGA	The Cancer Genome Atlas
TCIA	The Cancer Imaging Archiv
GBM	Glioblastoma multiforme
LGG	Low-grade glioma
MRI	Magnetic resonance imaging
mRNA	Messenger ribonucleic acid
microRNA	Micro ribonucleic acid
IPA	Ingenuity pathway analysis
*POSTN*	Periostin
KPS	Karnofsky performance status
VAK	Volume, Age, and KPS
*MGMT*	O6-methylguanine-DNA-methyltransferase
*EGFR*	Epidermal growth factor receptor
rCBVner	Relative cerebral blood volume of non-enhancing region
OS	Overall survival
PS	Permeability surface
*CBV*	Cerebral blood volume
*VEGF*	Vascular endothelial growth factor
*HIF1A*	Hypoxia-inducible factor 1-alpha
*VASH2*	Vasohibin-2
*C3*	Complement component 3
*AMOT*	Angiomotin
*NF1*	Neurofibromin 1
WHO	World health organization
rCBV	Relative cerebral blood volume
*PTEN*	Phosphatase and tensin homolog
*COL15A1*	Collagen alpha-1(XV) chain
*SPARC*	Secreted protein acidic and rich in cysteine
*ANPEP*	Alanyl aminopeptidase
*CBFB*	Core-binding factor subunit beta
*STRN*	Striatin
*TMED10*	Transmembrane P24 trafficking protein 10
*EGF*	Epithelial growth factor
*ARAF*	A-Raf proto-oncogene
*TRAF*	TNF receptor-associated factor 1
ROI	Region of interest
*GAP43*	Growth associated protein 43
*WWTR1*	WW domain-containing transcription regulator 1
*TP53*	Tumor protein P53
*PGC1alpha*	Peroxisome proliferator-activated receptor gamma coactivator 1-alpha
*PAR1*	Prader Willi/Angelman region RNA 1
*HOXC6*	Homeobox C6
*RB1*	Retinoblastoma protein
*PDGFRA*	Platelet-derived growth factor receptor A
AUC	Area under the curve
*HRAS*	Harvey rat sarcoma viral oncogene homolog
PARADIGM	Pathway recognition algorithm using data integration on genomic models
*VEGFR*	Vascular endothelial growth factor receptor
*Ang*	Angiopoietin
*Tie2*	Tyrosine-protein kinase receptor Tie-2
*WNT*	Wnt family member 1
*IRF9*	Interferon regulatory factor 9
*XRCC1*	X-ray cross-complementing
PsP	Pseudoprogression
TTP	True tumor progression
GSEA	Gene set enrichment analysis
PWI	Perfusion weighted imaging
*ANG*	Angiogenin
*TGFB2*	Transforming growth factor-beta 2
BEVR	Bounding ellipsoid volume ratio
BEDR1	Bounding ellipsoid diameter ratio 1
BEDR2	Bounding ellipsoid diameter ratio 2
MF	Margin fluctuation
ASD	Angular standard deviation
*AMKP*	AMP-activated protein kinase
*PI3k*	Phosphoinositide 3-kinase
*AKT*	Serine/threonine kinase 1
*mTOR*	Mechanistic target of rapamycin kinase
*NGF*	Nerve growth factor; Phosphatidylinositol-4,5-bisphosphate 3-kinase
*AKT*	RAC-alpha serine/threonine-protein kinase
*IL-8*	Interleukin 8
CGGA	Chinese glioma genome atlas
*TIMP1*	TIMP metallopeptidase inhibitor 1
*ROS1*	ROS proto-oncogene 1
*EREG*	Epiregulin
DCE	Dynamic contrast-enhanced
PET	Positron emission tomography
BPE	Breast parenchymal enhancement
GLCM	Gray-level-co-occurrence matrix
PR	Progesterone receptor
*CDK4*	Cyclin-dependent kinase 4
*BCL2*	B-cell lymphoma 2
ER	Estrogen receptor
BI-RADS	Breast imaging reporting and data system
*AR*	Androgen receptor
*ESR1*	Estrogen receptor 1
*IL6-*	Interleukin 6
*DDIT4*	DNA damage-inducible transcript 4
ccRCC	Clear cell renal cell carcinoma
pRCC	Papillary renal cell carcinoma
RCC	Renal cell carcinoma
VHL	Von Hippel–Lindau
CT	Computed tomography
*BAP1*	BRCA1 associated protein 1
*MUC4*	Mucin 4, Cell surface associated
ANN	Artificial neural network
RF	Random forest
*PBRM*	Polybromo-1
CLOVAR	Classification of ovarian cancer
CIN	Contrast-induced nephropathy
CER	Contrast-enhancing ratio
CEV	Contrast-enhancing volume
