# Supplementary material for: TCGA-TCIA Impact on Radiogenomics Cancer Research: A Systematic Review
Source: Int J Mol Sci. 2019 Nov 29;20(23):6033. doi: 10.3390/ijms20236033 (PMC6929079; doi:10.3390/ijms20236033)
Supplement: Supplementary file 1 [file ijms-20-06033-s001.pdf]

Table S1. Radiogenomic studies with TCGA/TCIA data.

First Author	Name	Journal	Year	Number of patients
**Glioblastoma Multiforme (GBM) and Low Grade Glioma (LGG)**				
Zinn et al [10]	Radiogenomic Mapping of Edema/Cellular Invasion MRI-Phenotypes in Glioblastoma Multiforme	Plos One	2011	78
Zinn et al [12]	A Novel Volume-Age-KPS (VAK) Glioblastoma Classification Identifies a Prognostic Cognate microRNA-Gene Signature	Plos One	2012	78
Jain et al [18]	Correlation of perfusion parameters with genes related to angiogenesis regulation in glioblastoma: a feasibility study.	AJNR Am J Neuroradiol	2012	45
Jain et al [19]	Genomic Mapping and Survival Prediction in Glioblastoma: Molecular Subclassification Strengthened by Hemodynamic Imaging Biomarkers	Radiology	2013	18
Gutman et al [22]	MR Imaging Predictors of Molecular Profile and Survival: Multi-institutional Study of the TCGA Glioblastoma Data Set	Radiology	2013	75
Gevaert et al [13]	Glioblastoma Multiforme:Exploratory Radiogenomic Analysis by Using Quantitative Image Features	Radiology	2014	55
Colen et al [15]	Imaging genomic mapping of an invasive MRI phenotype predicts patient outcome and metabolic dysfunction: a TCGA glioma phenotype research group project.	BMC Med Genomics	2014	92
Jain et al [23]	Outcome Prediction in Patients with Glioblastoma by Using Imaging, Clinical, and Genomic Biomarkers: Focus on the Nonenhancing Component of the Tumor	Radiology	2014	57
Rao et al [24]	A combinatorial radiographic phenotype may stratify patient survival and be associated with invasion and proliferation characteristics in glioblastoma.	J Neurosurg	2015	50
Gutman et al [21]	Somatic mutations associated with MRI-derived volumetric features in glioblastoma	Neuroradiology	2015	76
Nicolasjilwan et al [32]	Addition of MR imaging features and genetic biomarkers strengthens glioblastoma survival prediction in TCGA patients.	J Neuroradiol	2015	102
Colen et al [23]	Glioblastoma: imaging genomic mapping reveals sex-specific oncogenic associations of cell death	Radiology	2015	99
Itakura et al [26]	Magnetic resonance image features identify glioblastoma phenotypic subtypes with distinct molecular pathway activities	Science Translational Medicine	2015	144

Qian et al [27]	Identification of biomarkers for pseudo and true progression of GBM based on radiogenomics study.	Oncotarget	2016	38
Rao et al [20]	Integrative Analysis of mRNA, microRNA, and Protein Correlates of Relative Cerebral Blood Volume Values in GBM Reveals the Role for Modulators of Angiogenesis and Tumor Proliferation	Cancer Inform	2016	92
Heiland et al [21]	Molecular differences between cerebral blood volume and vessel size in glioblastoma multiforme	Oncotarget	2017	21
Liu et al [28]	Magnetic resonance perfusion image features uncover an angiogenic subgroup of glioblastoma patients with poor survival and better response to antiangiogenic treatment	Neuro Oncol	2017	48
Mazurowski et al [16]	Radiogenomics of lower-grade glioma: algorithmically-assessed tumor shape is associated with tumor genomic subtypes and patient outcomes in a multi-institutional study with The Cancer Genome Atlas data	Journal Of Neuro-oncology	2017	110
Lehrer et al [34]	Multiple-response regression analysis links magnetic resonance imaging features to de-regulated protein expression and pathway activity in lower grade glioma	Oncoscience	2017	57
Liu et al [29]	A radiomic signature as a non-invasive predictor of progression-free survival in patients with lower-grade gliomas	NeuroImage: Clinical	2018	84
Liao et al [30]	Machine-learning based radiogenomics analysis of MRI features and metagenes in glioblastoma multiforme patients with different survival time	J Cell Mol Med	2019	137
**Breast Cancer (BRCA)**				
Mazurowski et al [38]	Radiogenomic Analysis of Breast Cancer: Luminal B Molecular Subtype Is Associated with Enhancement Dynamics at MR Imaging	Radiology	2014	48
Guo et al [50]	Prediction of clinical phenotypes in invasive breast carcinomas from the integration of radiomics and genomics data.	J Med Imaging	2015	91
Zhu et al [51]	Deciphering Genomic Underpinnings of Quantitative MRI-based Radiomic Phenotypes of Invasive Breast Carcinoma.	Sci Rep	2015	91
Li et al [40]	Quantitative MRI radiomics in the prediction of molecular classifications of breast cancer subtypes in the TCGA/TCIA data set	NPJ Breast Cancer	2016	91
Li et al [41]	MR Imaging Radiomics Signatures for Predicting the Risk of	Radiology	2016	84

	Breast Cancer Recurrence as Given by Research Versions of MammaPrint, Oncotype DX, and PAM50 Gene Assays			
Wu et al [45]	Unsupervised clustering of quantitative image phenotypes reveals breast cancer subtypes with distinct prognoses and molecular pathways	Clinical Cancer Research	2018	96
Kim et al [46]	Associations between gene expression profiles of invasive breast cancer and breast imaging reporting and data system MRI lexicon	Annals of Surgical Treatment and Research	2017	70
Wu et al [49]	Heterogeneous Enhancement Patterns of Tumor-adjacent Parenchyma at MR Imaging Are Associated with Dysregulated Signaling Pathways and Poor Survival in Breast Cancer	Radiology	2017	126
Wu et al [39]	Identifying relations between imaging phenotypes and molecular subtypes of breast cancer: model discovery and external validation	Journal Of Magnetic Resonance Imaging	2017	84
Fan et al [48]	Tumour heterogeneity revealed by unsupervised decomposition of dynamic contrast-enhanced magnetic resonance imaging is associated with underlying gene expression patterns and poor survival in breast cancer patients.	Breast Cancer Res	2019	87
**Clear Cell Renal Cell Carcinoma (KIRC)**				
Shinagare et al [53]	Radiogenomics of clear cell renal cell carcinoma: preliminary findings of The Cancer Genome Atlas-Renal Cell Carcinoma (TCGA-RCC) Imaging Research Group.	Journal of Medical Imaging	2015	103
Bowen et al [56]	Radiogenomics of Clear Cell Renal Cell Carcinoma: Associations Between mRNA-Based Subtyping and CT Imaging Features	Academic Radiology	2018	177
**Other Tumours**				
Vargas et al [58]	Radiogenomics of High-Grade Serous Ovarian Cancer: Multireader Multi-Institutional Study from the Cancer Genome Atlas Ovarian Cancer Imaging Research Group	Radiology	2017	92
Lai et al [60]	Acute Tumor Transition Angle on Computed Tomography Predicts Chromosomal Instability Status of Primary Gastric Cancer: Radiogenomics Analysis from TCGA and Independent Validation	Cancers	2019	40
